# The secretome of stressed peripheral blood mononuclear cells increases tissue survival in a rodent epigastric flap model

**DOI:** 10.1002/btm2.10186

**Published:** 2020-09-22

**Authors:** Stefan Hacker, Rainer Mittermayr, Denise Traxler, Claudia Keibl, Annika Resch, Stefan Salminger, Harald Leiss, Philipp Hacker, Christian Gabriel, Bahar Golabi, Reinhard Pauzenberger, Paul Slezak, Maria Laggner, Michael Mildner, Wolfgang Michlits, Hendrik J. Ankersmit

**Affiliations:** ^1^ Division of Plastic and Reconstructive Surgery Medical University of Vienna Vienna Austria; ^2^ Christian Doppler Laboratory for Cardiac and Thoracic Diagnosis and Regeneration Vienna Austria; ^3^ Ludwig Boltzmann Institute for Experimental and Clinical Traumatology Vienna Austria; ^4^ Division of Rheumatology Medical University of Vienna Vienna Austria; ^5^ Department of Oral‐ and Maxillofacial Surgery University Clinic Sankt Poelten Sankt Poelten Austria; ^6^ Department of Blood Group Serology and Transfusion Medicine Medical University of Graz Austria; ^7^ Department of Dermatology Medical University of Vienna Vienna Austria; ^8^ Department of Plastic and Reconstructive Surgery Hospital Wiener Neustadt Wiener Neustadt Austria; ^9^ Division of Thoracic Surgery Medical University of Vienna Vienna Austria

**Keywords:** secretome, angiogenesis, flap surgery, necrosis, reconstructive surgery, tissue regeneration

## Abstract

Reconstructive surgery transfers viable tissue to cover defects and to restore aesthetic and functional properties. Failure rates after free flap surgery range from 3 to 7%. Co‐morbidities such as diabetes mellitus or peripheral vascular disease increase the risk of flap failure up to 4.5‐fold. Experimental therapeutic concepts commonly use a monocausal approach by applying single growth factors. The secretome of γ‐irradiated, stressed peripheral blood mononuclear cells (PBMCsec) resembles the physiological environment necessary for tissue regeneration. Its application led to improved wound healing rates and a two‐fold increase in blood vessel counts in previous animal models. We hypothesized that PBMCsec has beneficial effects on the survival of compromised flap tissue by reducing the necrosis rate and increasing angiogenesis. Surgery was performed on 39 male Sprague–Dawley rats (control, *N* = 13; fibrin sealant, *N* = 14; PBMCsec, *N* = 12). PBMCsec was produced according to good manufacturing practices (GMP) guidelines and 2 ml were administered intraoperatively at a concentration of 2.5 × 10^7^ cells/ml using fibrin sealant as carrier substance. Flap perfusion and necrosis (as percentage of the total flap area) were analyzed using Laser Doppler Imaging and digital image planimetry on postoperative days 3 and 7. Immunohistochemical stainings for von Willebrand factor (vWF) and Vascular Endothelial Growth Factor‐receptor‐3 (Flt‐4) were performed on postoperative day 7 to evaluate formation of blood vessels and lymphatic vessels. Seroma formation was quantified using a syringe and flap adhesion and tissue edema were evaluated clinically through a cranial incision by a blinded observer according to previously described criteria on postoperative day 7. We found a significantly reduced tissue necrosis rate (control: 27.8% ± 8.6; fibrin: 22.0% ± 6.2; 20.9% reduction, *p* = .053 vs. control; PBMCsec: 19.1% ± 7.2; 31.1% reduction, *p* = .012 vs. control; 12.9% reduction, 0.293 vs. fibrin) together with increased vWF+ vessel counts (control: 70.3 ± 16.3 vessels/4 fields at 200× magnification; fibrin: 67.8 ± 12.1; 3.6% reduction, *p* = .651, vs. control; PBMCsec: 85.9 ± 20.4; 22.2% increase, *p* = .045 vs. control; 26.7% increase, *p* = .010 vs. fibrin) on postoperative day 7 after treatment with PBMCsec. Seroma formation was decreased after treatment with fibrin sealant with or without the addition of PBMCsec. (control: 11.9 ± 9.7 ml; fibrin: 1.7 ± 5.3, 86.0% reduction, 0.004 vs. control; PBMCsec: 0.6 ± 2.0; 94.8% reduction, *p* = .001 vs. control; 62.8% reduction, *p* = .523 vs. fibrin). We describe the beneficial effects of a secretome derived from γ‐irradiated PBMCs on tissue survival, angiogenesis, and clinical parameters after flap surgery in a rodent epigastric flap model.

## 1 INTRODUCTION

Reconstructive surgery uses local, pedicled, or free flaps to restore the function and appearance after tumor resection or trauma.^1^, ^2^, ^3^ The rate of free flap failure ranges from 3 to 7%.^4^, ^5^ Patients requiring reconstructive surgery often suffer from diabetes mellitus or peripheral vascular disease, that were shown to increase the rate of flap failure up to 4.5‐fold.^6^, ^7^, ^8^, ^9^ This may lead to tissue necrosis and jeopardizes the reconstructive result. Necrosis can subsequently cause a series of events resulting in inflammation and further loss of tissue integrity.^10^ Experimental therapies including the use of antioxidants, vasodilators, anti‐inflammatory drugs, and hyperbaric oxygen led to a decrease in necrosis rates.^11^, ^12^, ^13^, ^14^, ^15^, ^16^ Alternatively, therapeutic angiogenesis through the application of pro‐angiogenic factors, such as Platelet‐derived growth factor (PDGF), and Vascular Endothelial Growth Factor (VEGF) has been demonstrated to improve the survival of compromised flaps by improving tissue perfusion.^10^, ^17^, ^18^, ^19^, ^20^, ^21^ The use of platelet‐rich plasma (PRP) resulted in an increase in flap survival of 20% by inducing angiogenesis and reducing the inflammatory response.^22^, ^23^ Currently, none of these therapies is used routinely during flap surgery. Fibrin sealants have been investigated for their hemostatic and adhesive properties and their ability to locally deliver and sustainably release growth factors, thus providing an important role as a biomatrix.^24^, ^25^, ^26^, ^27^, ^28^ Previous studies showed that γ‐irradiated, stressed peripheral blood mononuclear cells (PBMCs) represent an easily accessible source for the production of a cellular secretome (PBMCsec) with cytoprotective, regenerative, and immunomodulatory capacity, especially in ischemic tissues.^29^, ^30^ In contrast to experimental therapies applying only single growth factors, PBMCsec consists of a mixture of lipids, proteins, and extracellular vesicles that together represent the regenerative potential of this cell‐free therapy.^31^ Several mechanisms have already been characterized, indicating that the entire PBMCsec is required to exert its full action spectrum, as sub‐fractions thereof did not reach the same beneficial effects.^32^, ^33^ The pleiotropic effects of the secretome components better resemble the physiologic environment of wound healing occurring in the body, thus resulting in a faster and better regeneration. PBMCsec was successfully applied in animal models of wound healing, tissue ischemia or inflammation.^34^, ^35^, ^36^, ^37^, ^38^, ^39^ The application of PBMCsec enhanced wound healing and angiogenesis in a murine full‐thickness skin wound model. In vitro investigations showed increased migration and proliferation rates of keratinocytes, fibroblasts, and endothelial cells.^35^ In a porcine model of burn injury, PBMCsec significantly improved the epidermal thickness, and led to a two‐fold increase in angiogenesis.^36^ The safety and tolerability of topically administered autologous PBMCsec in human dermal wounds has already been proven in a clinical phase I trial (ClinicalTrials.gov Identifier: NCT02284360).^40^ An international, multi‐center, randomized, double‐blinded phase II clinical trial was recently initiated to investigate the regenerative effects of topically applied allogenic PBMCsec in chronic diabetic foot ulcers (ClinicalTrials.gov Identifier: NCT04277598, EudraCT number: 2018‐001653‐27). We hypothesized that the intraoperative application of PBMCsec leads to improved flap survival and increased angiogenesis. In contrast to previous experimental therapies, the pleiotropic effects of PBMCs resemble the physiologic wound healing environment resulting in increased regeneration, decreased inflammation, improved microvascular perfusion, and advanced angiogenesis.

## 2 RESULTS

### 2.1 Treatment with PBMCsec reduces flap necrosis

**FIGURE 1:**
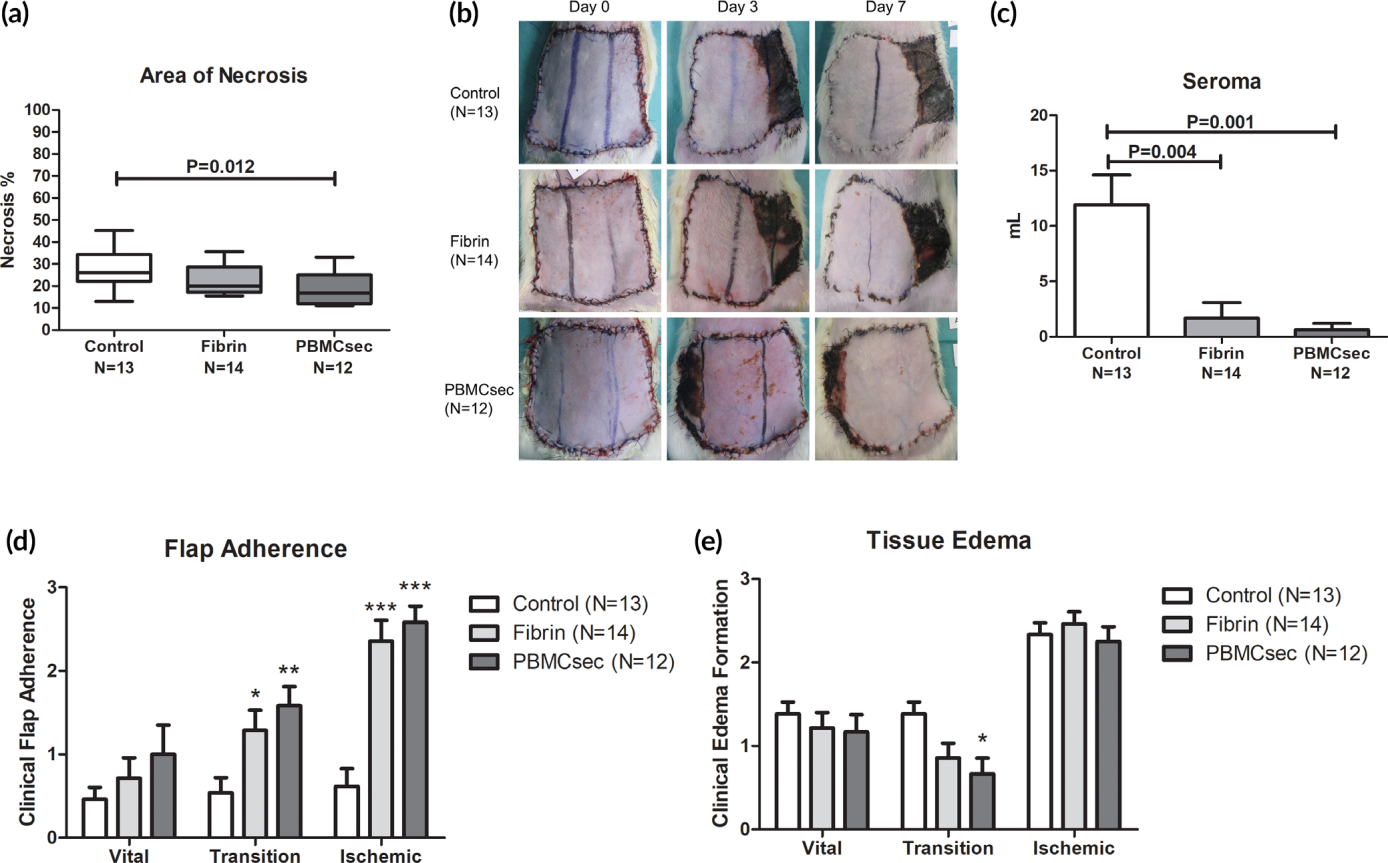
(a) The tissue necrosis rate was significantly reduced after a single, intraoperative application of the secretome derived from γ‐irradiated PBMCs (PBMCsec). Only the combinatory use of fibrin and PBMCsec showed significantly improved results. (b) Examples for the development of tissue necrosis over the postoperative period are shown for each group. (c) Seroma formation was evaluated on postoperative day 7. The use of fibrin sealant alone or in combination with PBMCsec significantly reduced the volume of seroma found in the surgical wounds. Results are given as mean ± SEM. (d) Flap adherence to the underlying tissue was evaluated clinically as parameter for tissue integration. Both groups, fibrin sealant alone and in combination with PBMC secretomes, showed markedly improved rates of flap adherence on postoperative day 7. (e) Tissue edema formation was comparable between all groups. However, in the transition zone of the flap, PBMCsec treatment led to significantly reduced clinical occurrence of edema 7 days after surgery. (*, <0.05 vs. control; **, <0.01 vs. control; ***, <0.001 vs. control)

The relative area of necrosis compared to the flap size was significantly reduced through the intraoperative application of PBMCsec. On day 7 after surgery, we found a complete demarcation of the necrotic area in the preoperatively defined ischemic area of the flap. Comparing the groups, the size of the necrotic areal was 27.8% ± 8.6 in the control group, 22.0% ± 6.2 in the fibrin group (20.9% reduction, *p* = .053 vs. control), and 19.1% ± 7.2 in the PBMCsec group (31.1% reduction, *p* = .012 vs. control; 12.9% reduction, 0.293 vs. fibrin). (Figure 1a,b, Table 1).

**TABLE 1:** Overview of all results

	Control (*N* = 13)	Fibrin (*N* = 14)	PBMCsec (*N* = 12)
*Flap necrosis (%)*			
Mean	27.8	22.0	19.1
*SD*	8.6	6.2	7.2
*p*‐value		.053 vs. control	.012 vs. control .293 vs. fibrin
*Seroma (ml)*			
Mean	11.9	1.7	0.6
*SD*	9.7	5.3	2.0
*p*‐value		.004 vs. control	.001 vs. control .523 vs. fibrin
*Flap adherence*			
*Vital zone*			
Mean	0.46	0.71	1.00
*SD*	0.52	0.91	1.21
*p*‐value		.685 vs. control	.437 vs. control .631 vs. fibrin
*Transition zone*			
Mean	0.54	1.29	1.58
*SD*	0.66	0.91	0.79
*p*‐value		.038 vs. control	.004 vs. control .432 vs. fibrin
*Ischemic zone*			
Mean	0.62	2.36	2.58
*SD*	0.77	0.93	0.67
*p*‐value		<.001 vs. control	<.001 vs. control .631 vs. fibrin
*Tissue edema*			
*Vital zone*			
Mean	1.38	1.21	1.17
*SD*	0.51	0.70	0.72
*p*‐value		.616 vs. control	.538 vs. control .899 vs. fibrin
*Transition zone*			
Mean	1.38	0.86	0.67
*SD*	0.51	0.66	0.65
*p*‐value		.068 vs. control	.016 vs. control .527 vs. fibrin
*Ischemic zone*			
Mean	2.33	2.46	2.25
*SD*	0.49	0.52	0.62
*p*‐value		.611 vs. control	.843 vs. control .470 vs. fibrin
*Flap perfusion (PU)*			
*Transition zone*			
*Preoperative*			
Mean	466.9	448.6	463.0
*SD*	113.6	75.5	88.1
*Postoperative day 3*			
Mean	525.9	564.5	612.6
*SD*	140.5	86.5	217.1
*p*‐value	.292 vs. pre	.001 vs. pre	.026 vs. pre
*Postoperative day 7*			
Mean	664.6	777.8	726.4
*SD*	229.6	170.6	183.7
*p*‐value	.032 vs. pre .046 vs. day 3	<.001 vs. pre <.001 vs. day 3	.003 vs. pre .186 vs. day 3
*Ischemic zone*			
*Preoperative*			
Mean	427.6	400.5	439.1
*SD*	100.8	46.0	70.0
*Postoperative day 3*			
Mean	135.1	123.1	138.6
*SD*	46.2	41.5	63.3
*p*‐value	<.001 vs. pre	<.001 vs. pre	<.001 vs. pre
*Postoperative day 7*			
Mean	174.1	198.9	257.6
*SD*	83.4	144.0	154.7
*p*‐value	<.001 vs. pre .038 vs. day 3	<.001 vs. pre .057 vs. day 3	.007 vs. pre .017 vs. day 3
*vWF+ blood vessels/4 fields (N)*			
Mean	70.3	67.8	85.9
*SD*	16.3	12.1	20.4
*p*‐value		.651 vs. control	.045 vs. control .010 vs. fibrin
*Flt‐4+ cells/4 fields (N)*			
Mean	98.2	74.5	111.0
*SD*	53.2	46.0	66.2
*p*‐value		.227 vs. control	.597 vs. control .112 vs. fibrin

### 2.2 Treatment with fibrin sealant and PBMCsec markedly reduces postoperative seroma formation

The mean seroma volume was significantly higher in the control group (11.9 ml ± 9.7) compared to the fibrin (1.7 ml ± 5.3, 86.0% reduction, 0.004 vs. control) and the PBMCsec group (0.6 ml ± 2.0, 94.8% reduction, *p* = .001 vs. control; 62.8% reduction, *p* = .523 vs. fibrin). (Figure 1c, Table 1) Consequently, the extent of flap adherence was considerably improved in both treatment groups, especially in the clinically relevant transition and ischemic zones. In the vital zone, the extent of flap adherence was 0.46 ± 0.52 in the control group, 0.71 ± 0.91 in the fibrin group (54.3% increase, *p* = .685 vs. control), and 1.00 ± 1.21 in the PBMCsec group (117.4% increase, *p* = .437 vs. control; 40.8% increase, *p* = .631 vs. fibrin). In the transition zone, the extent of flap adherence was 0.54 ± 0.66 in the control group, 1.29 ± 0.91 in the fibrin group (138.9% increase, *p* = .038 vs. control), and 1.58 ± 0.79 in the PBMCsec group (192.6% increase, *p* = .004 vs. control; 22.5% increase, *p* = .432 vs. fibrin) In the ischemic zone, the extent of flap adherence was 0.62 ± 0.77 in the control group, 2.36 ± 0.93 in the fibrin group (280.6% increase, *p* < .001 vs. control), and 2.58 ± 0.67 in the PBMCsec group (316.1% increase, *p* < .001 vs. control; 9.3% increase, *p* = .631 vs. fibrin; Figure 1d, Table 1) In the transition zone of the flap, the amount of tissue edema was shown to be significantly less pronounced in the PBMCsec group compared to the control group. In the vital zone, the extent of tissue edema was 1.38 ± 0.51 in the control group, 1.21 ± 0.70 in the fibrin group (12.3% reduction, *p* = .616 vs. control), and 1.17 ± 0.72 in the PBMCsec group (15.2% reduction, *p* = .538 vs. control; 3.3% reduction, 0.899 vs. fibrin). In the transition zone, the extent of tissue edema was 1.38 ± 0.51 in the control group, 0.86 ± 0.66 in the fibrin group (37.7% reduction, 0.068 vs. control), and 0.67 ± 0.65 in the PBMCsec group (51.4% reduction, *p* = .016 vs. control; 22.1% reduction, *p* = .527 vs. fibrin). In the ischemic zone, the extent of tissue edema was 2.33 ± 0.49 in the control group, 2.46 ± 0.52 in the fibrin group (5.6% increase, *p* = .611 vs. control), and 2.25 ± 0.62 in the PBMCsec group (3.4% reduction, 0.843 vs. control; 8.5% reduction, *p* = .470 vs. fibrin; Figure 1e, Table 1).

### 2.3 Flap perfusion only partially recovers to preoperative values in the ischemic zone

**FIGURE 2:**
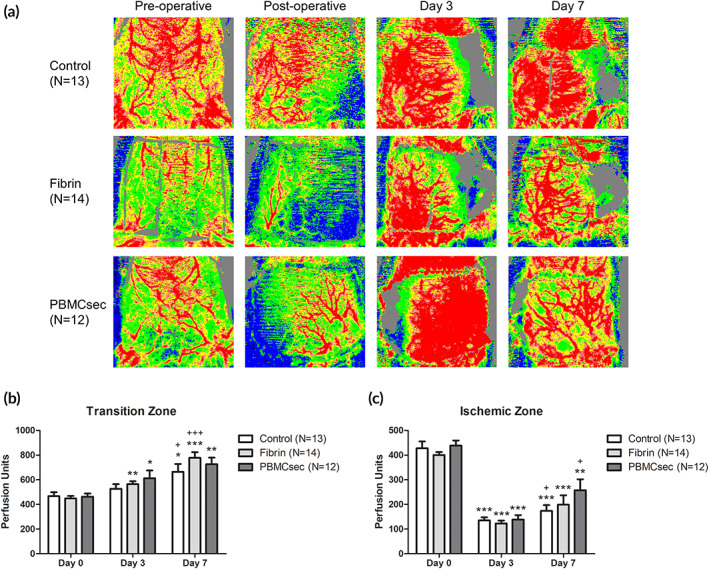
(a) Flap perfusion was measured using Laser Doppler Imaging (LDI). Examples for the color‐coded image after perfusion measurements are given for each group. After ligation of the unilateral neurovascular bundle, the perfusion decreases on the contralateral side. (b) In the transition zone of the flap, a constant progression of tissue perfusion was found in all study groups. (c) After ligation of the contralateral inferior epigastric vessels, we evidenced a steep decline in tissue perfusion in the ischemic zone of the flap. The LDI measurements showed a significant recovery on postoperative day 7. The strongest effect was observed in the PBMCsec treated animals. (*, <0.05 vs. day 0; **, <0.01 vs. day 0; ***, <0.001 vs. day 0; +, <0.05 vs. day 3; ++, <0.01 vs. day 3; +++, <0.001 vs. day 3; results are given as mean ± SEM)

In the transition zone of the flap, the perfusion units measured by LDI showed a steady increase from preoperative values to postoperative day 7. The strongest change overall was shown in the fibrin group, whereas the increase was markedly weaker in the control group. In the control group, LDI values (perfusion units) were 466.9 ± 113.6 preoperatively, 525.9 ± 140.5 on postoperative day 3 (12.7% increase, *p* = .292 vs. preoperative), and 664.6 ± 229.6 on postoperative day 7 (42.3% increase, *p* = .032 vs. preoperative; 26.4% increase, *p* = .046 vs. day 3). In the fibrin group, LDI values were 448.6 ± 75.5 preoperatively, 564.5 ± 86.5 on postoperative day 3 (25.1% increase, *p* = .001 vs. preoperative), and 777.8 ± 170.6 on postoperative day 7 (73.3% increase, *p* < 0.001 vs. preoperative; 37.8% increase, *p* < 0.001 vs. day 3). In the PBMCsec group, LDI values were 463.0 ± 88.1 preoperatively, 612.6 ± 217.1 on postoperative day 3 (32.3% increase, *p* = .026 vs. preoperative), and 726.4 ± 183.7 on postoperative day 7 (56.9% increase, *p* = .003 vs. preoperative; 18.6% increase, *p* = .186 vs. day 3) (Figure 2a,b, Table 1).

The situation appeared completely different in the ischemic zone. The decline from preoperative to postoperative values on day 3 was steep in all study groups. Here, the recovery of the flap perfusion was clearly stronger in the PBMCsec group. In the control group, LDI values (perfusion units) were 427.6 ± 100.8 preoperatively, 135.1 ± 46.2 on postoperative day 3 (68.4% reduction, *p* < .001 vs. preoperative), and 174.1 ± 83.4 on postoperative day 7 (59.3% reduction, *p* < .001 vs. preoperative; 28.9% increase, *p* = .038 vs. day 3). In the fibrin group, LDI values were 400.5 ± 46.0 preoperatively, 123.1 ± 41.5 on postoperative day 3 (69.2% reduction, *p* < 0.001 vs. preoperative), and 198.9 ± 144.0 on postoperative day 7 (50.3% reduction, *p* < 0.001 vs. preoperative; 61.6% increase, *p* = .057 vs. day 3). In the PBMCsec group, LDI values were 439.1 ± 70.0 preoperatively, 138.6 ± 63.3 on postoperative day 3 (68.4% reduction, *p* < 0.001 vs. preoperative), and 257.6 ± 154.7 on postoperative day 7 (41.3% reduction, *p* = .007 vs. preoperative; 85.9% increase, *p* = .017 vs. day 3) (Figure 2a,c, Table 1).

### 2.4 Blood vessel density is increased in the PBMCsec treated flaps

**FIGURE 3:**
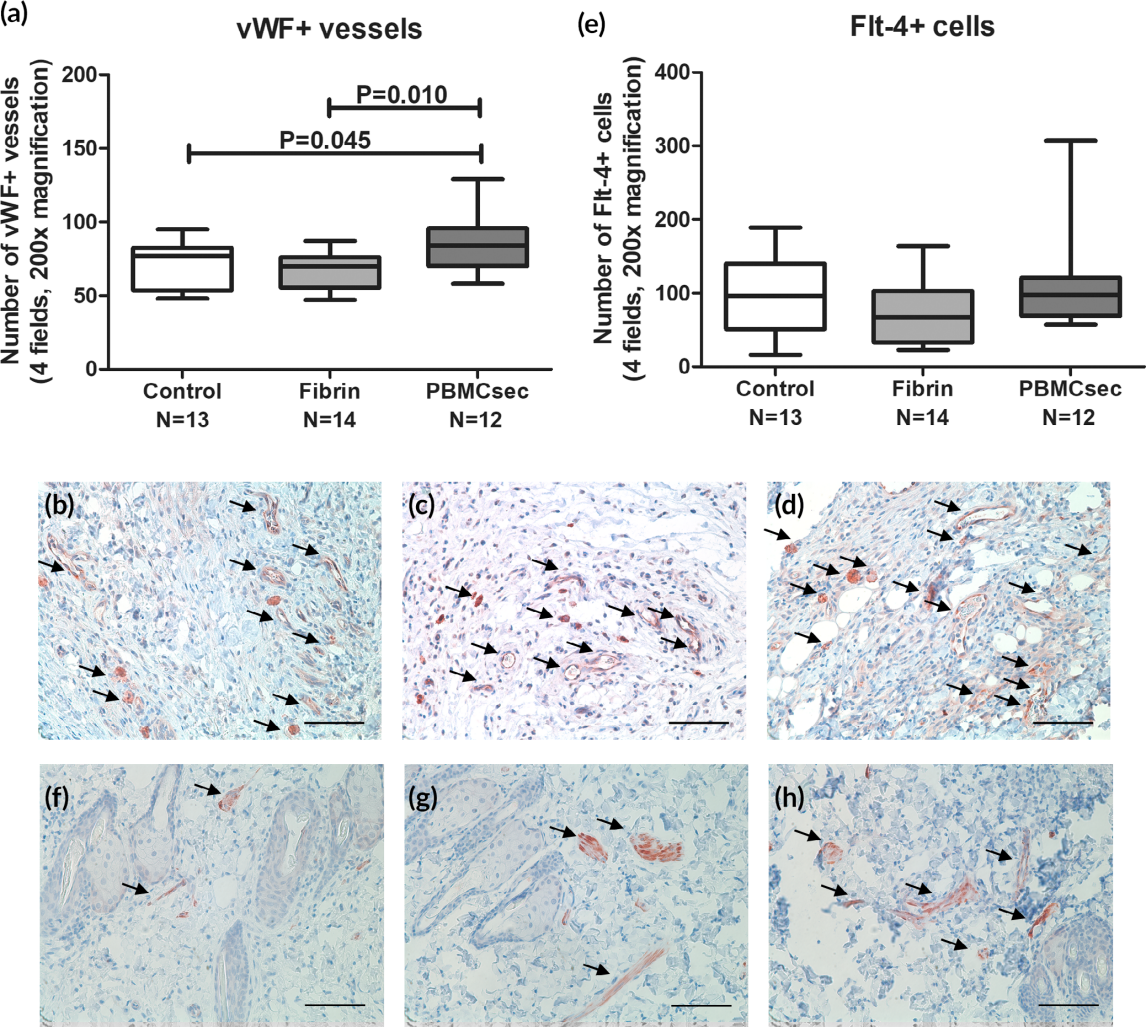
(a) The number of vWF+ blood vessels was significantly increased in the flaps treated with PBMCsec. Fibrin sealant alone did not affect blood vessel density compared to the control group. These findings are in accordance with previously published results of pro‐angiogenic effects in other animal models.^35^, ^36^ Representative stainings for vWF of control (b), fibrin (c), and PBMCsec (d) treated animals are shown. (Scale Bar = 100 μm, arrows indicate vWF+ blood vessels) (e) Flt‐4 stainings were performed to determine the effect on lymphangiogenesis in the flap tissue. We did not evidence any alterations of the Flt‐4+ cell counts between the study groups. Representative stainings for Flt‐4 of control (f), fibrin (g), and PBMCsec (h) treated animals are shown. (Scale Bar = 100 μm, arrows indicate Flt‐4+ cells)

We found a statistically significant increase in blood vessels in the PBMCsec group (85.9 ± 20.4 vessels/4 fields at 200× magnification; 22.3% increase, *p* = .045 vs. control; 26.8% increase, *p* = .010 vs. fibrin) compared to both, the control (70.3 ± 16.3) and the fibrin (67.8 ± 12.1; 3.6% reduction, *p* = .651 vs. control) group. (Figure 3a–d, Table 1) No difference was found in the presence of lymphatic vessels determined by cells staining positive for VEGFR‐3 (control: 98.2 ± 53.2 cells/4 fields at 200× magnification; fibrin: 74.5 ± 46.0; 24.1% reduction, *p* = .227 vs. control; PBMCsec: 111.0 ± 66.2; 13.0% increase, *p* = .597 vs. control; 49.0% increase, *p* = .112 vs. fibrin; Figure 3e–h, Table 1).

## 3 DISCUSSION

We were able to demonstrate a reduced postoperative flap necrosis and a significantly improved rate of angiogenesis after flap surgery through the application of secretome derived from γ‐irradiated, stressed PBMCs in combination with fibrin glue. Additionally, an improved flap adherence and reduced postoperative seroma formation were observed. The critical ischemia of the contralateral flap areas mimics the clinical situation in patients with impaired flap perfusion.^7^, ^41^, ^42^ Fibrin sealant is commercially available and was previously used as a carrier substance for growth factors and stem cells in animal flap models.^24^, ^26^, ^43^ It has gained widespread acceptance in clinical use.^28^, ^44^, ^45^, ^46^ The combination of PBMCsec at the described concentration of 2.5 × 10^7^ cells/ml and fibrin glue may improve the flap survival rate in patients with co‐morbidities who are at an increased risk for postoperative flap failure. Cost‐effectiveness would be achievable through the prevention of secondary surgeries. Previous results attested the consistency of the PBMCsec production process regarding its composition, content, and stability.^47^ Its safety and tolerability in dermal wounds have been proven in a clinical phase I trial.^40^, ^48^ PBMCsec was categorized as a biological medicinal product (Directive 2001/83/EC) by the regulatory authorities. A clinical phase II trial has been initiated and the approval for clinical use could be reachable within the next years. The proposed phase II clinical study is primarily based on the regulatory requirements of EMA/CHMP/ICH/731268/1998 and EMA/CMPM/ICH/286/1995.^48^ Limitations of this study are the use of a small animal model. Further studies are needed to show the effects on flap necrosis in large animal models and humans. In addition, the production of PBMCsec requires an adequate infrastructure to ensure the quality needed to achieve the described results.

**FIGURE 4:**
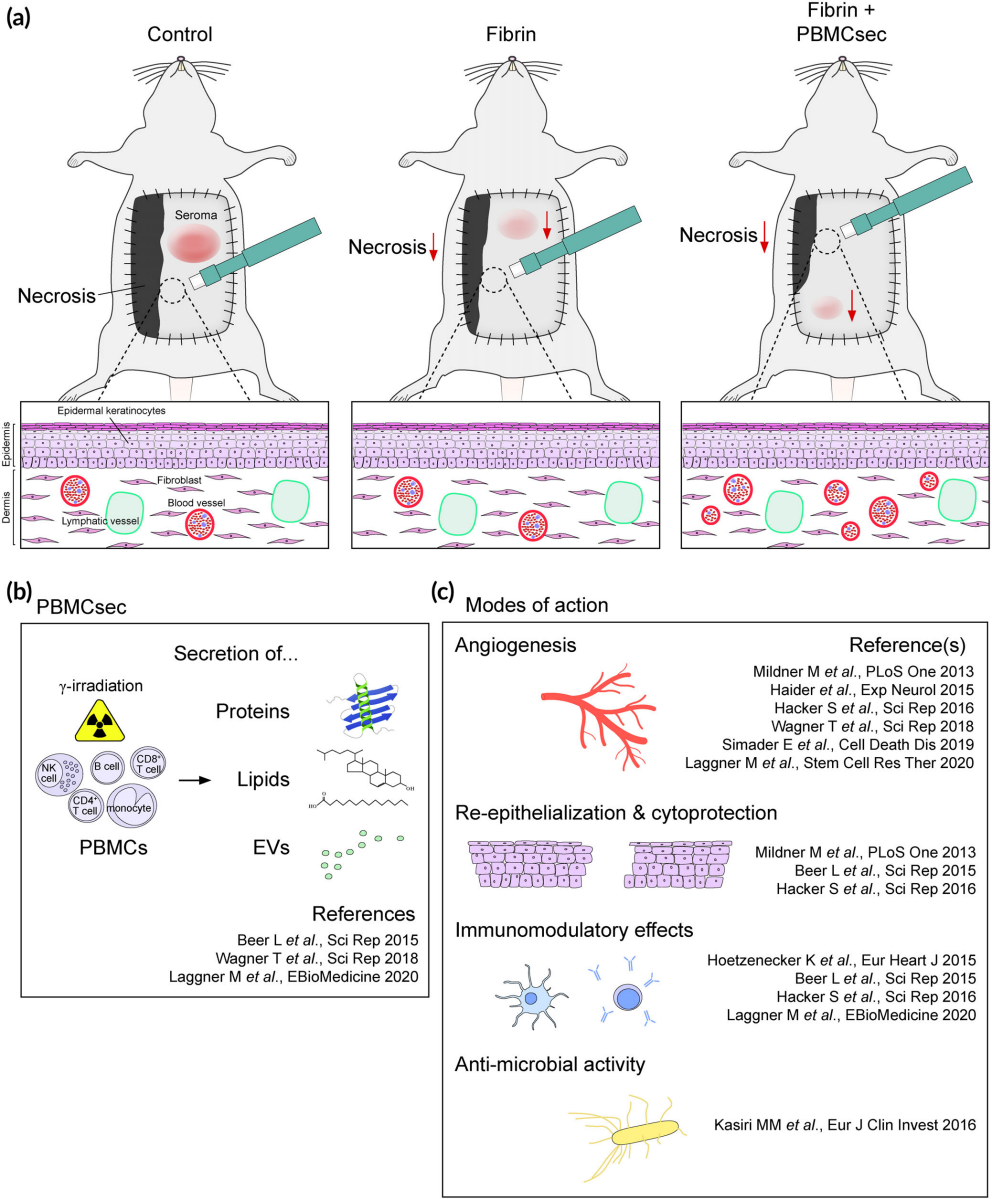
The proposed method of action for the use of PBMCsec in flap surgery is shown. (a) The intraoperative application of PBMCsec leads to a reduced rate of flap necrosis and seroma. In the tissue biopsies taken from the border between viable and necrotic tissue, increased rates of angiogenesis were found. (b) The irradiation of PBMC causes a switch toward a regenerative phenotype and induces the secretion of proteins, lipids, and EVs, which form the secretome. (c) PBMCsec resembles the physiologic environment of wound healing and regeneration and therefore has pleiotropic effects

PBMCsec comprises a plethora of biologically active components including proteins, lipids, and extracellular vesicles and the mechanisms of action have been the focus of extensive research over the past years.^30^, ^31^, ^33^ On a cellular level, the irradiation of PBMCs augments the release of extracellular vesicles and shifts the protein, lipid, and miRNA composition of the secretome toward a regenerative phenotype.^33^ In most of the studied biological processes the integrity of the entire PBMC secretome was necessary to achieve full regenerative effects as sub‐fractions or single cell types did not reach the same capacity.^32^, ^33^ Unlike previously described monocausal therapies, the effects of PBMCsec can therefore be attributed to its heterogeneity, resembling the physiological environment necessary for tissue regeneration. Previously described therapies with single growth factors significantly reduced postoperative flap necrosis at a similar rate to the described results. However, these experimental therapies mainly focused on angiogenesis and therefore lack additional immunomodulatory and cytoprotective effects that may play a role in patients with co‐morbidities.^26^, ^27^ In correlation with the previously described effects in animal models of wound healing we found an increased density of vWF+ blood vessels after PBMCsec treatment.^35^, ^36^ The TNF/TNFRSF1B signaling pathway was described as the mechanism underlying the γ‐irradiation‐induced pro‐angiogenic activity of PBMCsec.^32^ Clinically, the impairment of flap perfusion leads to inflammation and secondary tissue damage.^10^, ^49^ The immunomodulatory and cytoprotective effects of PBMCsec were previously described in animal models of myocarditis, contact hypersensitivity, and cerebral ischemia.^37^, ^50^, ^51^ The reduction of postischemic inflammatory reactions may have a beneficial effect on tissue viability. The clinically relevant bactericidal activity of PBMCsec was mainly attributed to the high abundance of different antimicrobial peptides in the secretome.^52^ In combination with reduced seroma formation, improved tissue edema and flap adherence, the angiogenic, cytoprotective, antibacterial, and immunosuppressive properties of PBMC secretome may therefore play an additional role in the markedly increased flap survival after surgery (Figure 4).

## 4 MATERIALS AND METHODS

### 4.1 Ethics statement

**FIGURE 5:**
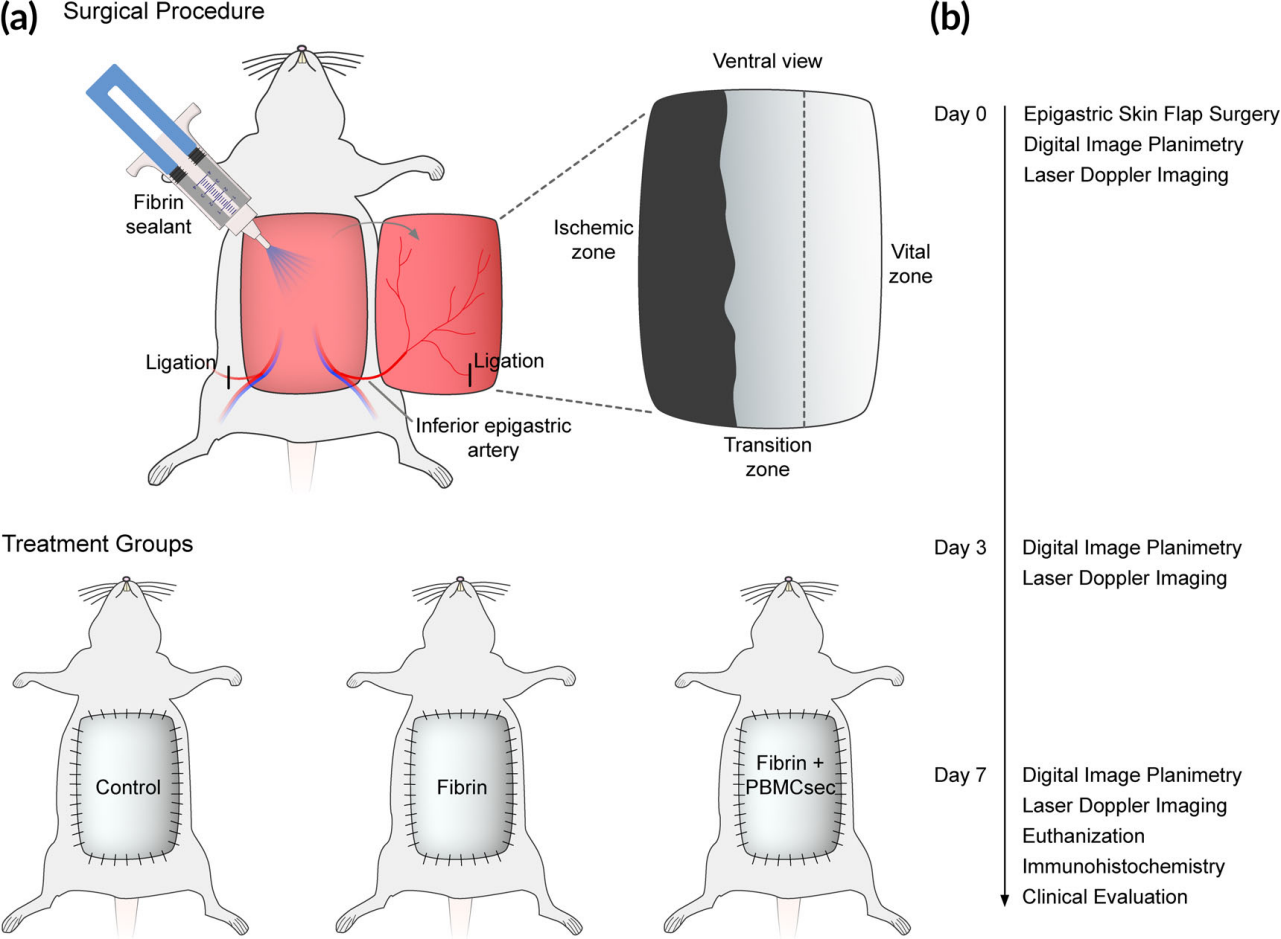
The study design and timeline are shown. Ligation of the unilateral inferior epigastric artery leads to a controlled necrosis of the contralateral side of the flap. Measurements were performed preoperatively, postoperatively, and on postoperative days 3 and 7

All animal experiments were approved by the local ethical authority of Vienna (807408/2013) and met the institutional and national guidelines for the use and care of laboratory animals. The local ethics committee at the Medical University of Vienna (2010/034) approved blood donations by healthy volunteers. A detailed description of the study design is depicted in Figure 5.

### 4.2 Production of secretome

PBMCsec was produced according to previously described methods (LOT: 399014).^39^ In anticipation of possible future clinical applications, the production followed GMP guidelines. This production process was shown to yield reproducible results.^47^ Human PBMCs were isolated from the whole blood of healthy donors after obtaining informed consent by density gradient centrifugation. After 60‐Gray γ‐irradiation, the cells were cultivated for 24 hr with CellGenix GMP DC medium (Cellgenix, Freiburg, Germany) at a concentration of 2.5 × 10^7^ cells/ml under sterile conditions. After centrifugation, the cells were discarded and the supernatant was collected and filtrated. For viral inactivation, the supernatant was treated with methylene blue (MB) plus light treatment using the Theraflex MB‐Plasma system, the Theraflex MB‐Plasma bag system, and an LED‐based illumination device (MacoPharma, Langen, Germany). MB and photoproducts were removed by consecutive Blueflex filtration steps. After lyophilization of the viral‐inactivated cell culture supernatant, the lyophilized powder was treated with γ‐irradiation to further reduce the risk of viral contamination. The lyophilized supernatant of apoptotic PBMCs was considered pathogen‐free after this two‐step process and was stored at −80°C until re‐suspension as needed using sterile water (Aqua ad injectabilia, B Braun, Melsungen, Germany).

### 4.3 Rodent epigastric flap model

**FIGURE 6:**
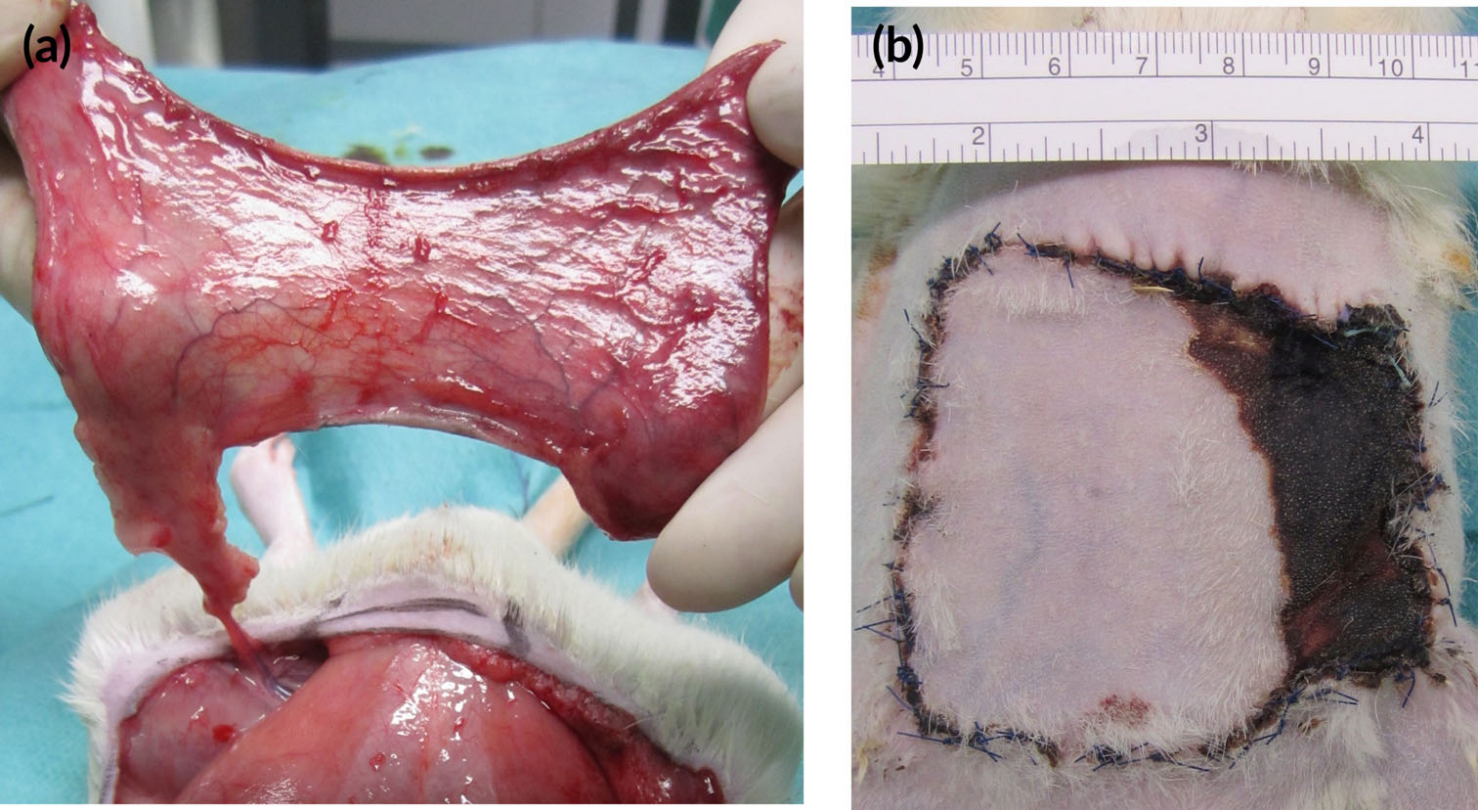
(a) A previously described epigastric flap model was used to evaluate the regenerative and angiogenic effects of the treatment protocol.^42^ After the ligation of the contralateral vascular bundle, the flap pedicle consists only of the unilateral inferior epigastric vessels. (b) The ligation of one neurovascular bundle leads to the controlled necrosis of the contralateral side of the flap tissue

The rodent epigastric flap model was performed as described.^42^ 39 adult male Sprague–Dawley rats (weighing 422 ± 30 g) were divided in three groups according to a computerized randomization protocol (surgery without additional treatment/“control,” *N* = 13; treatment with fibrin sealant/“fibrin,” *N* = 14; treatment with fibrin sealant containing PBMCsec/“PBMCsec,” *N* = 12). Animals were box‐induced using isoflurane and maintained under general anesthesia using ketamine and xylazine. The abdomen of each animal was shaved and depilated. Animals were placed in supine position on a surgical heating pad. The borders of the epigastric skin flap were marked using previously described anatomical landmarks: cranially the xiphoid, caudally the pubic region and bilaterally the distinct transition from the thin ventral skin to the coarse dorsal skin. This results in a flap size of approximately 8 × 8 cm.^42^ The flap was then divided into three distinct vertical zones: Vital zone (origin of supplying neurovascular bundle), transition zone (transition from adequate to insufficient vascular supply), and ischemic zone (area of dissected neurovascular bundle). An extended epigastric adipocutaneous flap was raised superficial to the abdominal muscular fascia from cranial to caudal. To render the contralateral part of the flap ischemic, either the left or the right inferior epigastric neurovascular bundle (according to the study randomization protocol) was ligated. The flap was sutured back to its native anatomical orientation. (Figure 6a,b) After completion of the surgical procedure, animals received analgesic therapy by subcutaneous administration of 0.05 mg/kg BW buprenorphin every 8 hr for 3 days. To prevent auto‐cannibalization, the incisor teeth in the upper and lower jaw were abraded under anesthesia on the day of surgery and during dressing changes. Three and seven days postoperatively, all animals were re‐anesthetized by isoflurane, digital images of the epigastric flaps were taken and the flaps were scanned using the Laser Doppler Imaging system (LDI, Moor LDI™, Moor Instruments Ltd., Devon, UK). On day 7, animals were euthanized by an overdose of pentobarbital, flaps were evaluated macroscopically, and full‐thickness adipocutaneous biopsies were obtained for immunohistological analyses.

### 4.4 Treatment protocol

Intraoperatively, the treatment of the animals was performed using commercially available fibrin sealants (ARTISS, Baxter, Deerfield, IL, USA) as carrier substance to locally administer the dissolved freeze‐dried supernatants. In the PBMCsec group, the secretome of 5 × 10^7^ cells mixed into 2 ml (final concentration: 2.5 × 10^7^ cells/ml) of fibrin sealant was applied evenly over the wound area between the flap and the abdominal muscular wall immediately before wound closure. In the fibrin sealant group, no PBMCsec was added to the fibrin sealant. Animals in the control group received no additional treatment during surgery.

### 4.5 Digital image planimetry

The surface area of the flap including the necrotic flap area was traced onto a transparent acrylic foil, which was then photographed. Digital images were analyzed using the ImageJ software.^53^ Measurements were performed after the surgery, on postoperative days 3 and 7. The entire flap surface area was defined by the flap borders and the flap necrosis rate was expressed as percentage of the total area. Flap areas which appeared black with hair loss, induration, and loss of skin elasticity were defined as necrotic.

### 4.6 Flap perfusion measurements

The flap perfusion was measured with the LDI system preoperatively, postoperatively, and on postoperative days 3 and 7. A low intensity (2 mW) laser light beam (wavelength 632.8 nm) scans the surface of the epigastric flap skin and generates a 2‐dimensional image of flap perfusion. Moving blood cells shift the frequency of the laser light according to the Doppler principle. This effect is converted into a color‐coded image. The LDI scan modus was set at 10 ms/pixel, and the resolution at 256 × 256 pixels. All laser scans were performed without skin contact at a standardized working distance of 20 cm. Flap perfusion was expressed in arbitrary perfusion units (PU) by the analysis software (Moor Instruments Ltd.). Perfusion was calculated as the mean of the PU for each of the 3 vertical zones: Vital zone, transition zone, and ischemic zone.

### 4.7 Macroscopic evaluation

Flaps were evaluated clinically on day 7 by a blinded observer. After a cranial incision, assessments were performed for seroma volume, flap adherence to the wound bed (0 = no adherence of the flap to the underlying abdominal wall, 1 = less than 50% of the flap area is adherent to the abdominal wall, 2 = 50% to less than 100% of the flap area is adherent to the abdominal wall, 3 = full adherence of the flap to the abdominal wall) and edema formation using a grading scale (0 = no visible edema, 1 = minimal signs of edema of the flap, 2 = moderate edema of the flap, 3 = maximum edema of the entire flap) as established previously (Table 2).^42^ Seroma fluid between the muscular abdominal wall and the flap was aspirated with a syringe and measured.

**TABLE 2:** The clinical scoring systems for flap adherence and tissue edema

	Flap adherence	Tissue edema
Grading		
0	No adherence of the flap to the underlying abdominal wall	No visible edema
1	Less than 50% of the flap area is adherent to the abdominal wall	Minimal signs of edema of the flap
2	50% to less than 100% of the flap area is adherent to the abdominal wall	Moderate edema of the flap
3	Full adherence of the flap to the abdominal wall	Maximum edema of the entire flap

### 4.8 Immunohistochemistry

Full‐thickness sections from epigastric skin flaps (border from vital to necrotic area) were used for immunohistochemical (IHC) analyses. IHC staining of vascular endothelial cells was performed using an antibody against von Willebrand Factor (vWF; Agilent, Santa Clara, CA) to visualize blood vessels. To evaluate the formation of lymphatic vessels, the tissue sections were also stained for VEGF‐receptor‐3 (Flt‐4, Santa Cruz Biotechnology, Dallax, TX). Staining was performed on paraffin‐embedded tissues after antigen retrieval by boiling in citrate‐buffer (pH = 6, Dako, Glostrup, Denmark) in a microwave for 5 min. After blocking the sections with 10% normal goat serum for 1 hr, the slides were incubated overnight in a humidified chamber at 4°C with the antibodies or isotype‐matched control (Abcam) antibody diluted in PBS containing 2% bovine serum albumin (BSA) and 10% goat serum. To visualize the stainings, sections were incubated with a horseradish peroxidase‐linked secondary antibody in PBS containing 2% BSA and 10% normal goat serum for 1 hr, followed by incubation with DAB Chromogen tablets (Dako). After washing, nuclear staining was performed by incubation with hematoxylin for 10 s. Slides were mounted with Fluoprep (bioMérieux, Marcy l'Etoile, France). vWF‐positive vessels were counted in four fields (200× magnification) per slide by a blinded observer. Flt‐4‐positive cells were quantitatively analyzed in four fields (200× magnification) by tissue cytometry using the HistoQuest™ software (TissueGnostics, Vienna, Austria).

### 4.9 Statistical analysis

We used IBM SPSS Statistics 24.0 (IBM, Armonk, NY) for data analysis. In case of a normal distribution of a metric variable, the Student's *t*‐test was used to compare groups. Otherwise, the nonparametric Mann–Whitney U‐Test was used. If not stated otherwise, results are given as mean ± standard deviation (SD). In all calculations, a *p*‐value <.05 was considered statistically significant. The *p*‐values were not adjusted for multiple comparisons.

## 5 CONCLUSIONS

In conclusion, we demonstrated a significantly reduced necrosis rate in combination with increased blood vessel density after a single, intraoperative application of the secretome derived from γ‐irradiated, stressed PBMCs in a rodent epigastric flap model. GMP‐compliant production of PBMCsec yields a consistent composition of the cell‐derived secretome as a biological medicinal product and recent studies have demonstrated its safety and tolerability.^40^, ^47^, ^48^ Our study identified a beneficial combinatory effect of PBMCsec and fibrin glue for flap survival of marginally perfused tissue areas, which might therefore represent a powerful tool for reconstructive and aesthetic surgeons.

## 6 CONFLICT OF INTEREST

The Medical University of Vienna has claimed financial interest and Hendrik J. Ankersmit holds patents related to this work and is a shareholder of Aposcience AG. All other authors declare no potential conflict of interest.
